# The Relationship Between Nurse’s Professional Quality of Life, Mindfulness, and Hardiness: A Cross-Sectional Study During the COVID-19 Outbreak

**DOI:** 10.3389/fpsyg.2022.866038

**Published:** 2022-07-11

**Authors:** Mohammad Ali Zakeri, Fatemeh Ghaedi-Heidari, Elham Khaloobagheri, Sayed Mortaza Hossini Rafsanjanipoor, Hamid Ganjeh, Hassan Pakdaman, Mitra Abbasifard, Mehdi Mehdizadeh, Abbas Zakeri Bazmandeh, Mahlagha Dehghan

**Affiliations:** ^1^Social Determinants of Health Research Center, Rafsanjan University of Medical Sciences, Rafsanjan, Iran; ^2^Non-Communicable Diseases Research Center, Rafsanjan University of Medical Sciences, Rafsanjan, Iran; ^3^Faculty of Nursing and Midwifery, Isfahan University of Medical Sciences, Isfahan, Iran; ^4^Department of Internal Surgery Nursing, Ali-Ibn Abi-Talib Hospital, Rafsanjan University of Medical Sciences, Rafsanjan, Iran; ^5^Department of Nursing, Ali-Ibn Abi-Talib Hospital, Rafsanjan University of Medical Sciences, Rafsanjan, Iran; ^6^Department of Internal Medicine, Ali-Ibn Abi-Talib Hospital, School of Medicine, Rafsanjan University of Medical Sciences, Rafsanjan, Iran; ^7^Department of Medical Nanotechnology, School of Advanced Medical Sciences and Technologies, Shiraz University of Medical Sciences, Shiraz, Iran; ^8^Department of Critical Care Nursing, Nursing Research Center, Kerman University of Medical Sciences, Kerman, Iran

**Keywords:** COVID-19, hardiness, mindfulness, nurses, professional quality of life

## Abstract

**Background:**

In the pandemic of Coronavirus Disease 2019 (COVID-19) disease, various factors, such as workplace factors, and psychological variables, can affect the occupational status of nurses. This study aimed to assess the relationship between nurses’ professional quality of life, mindfulness, and hardiness during the outbreak of COVID-19.

**Methods:**

This cross-sectional study included 239 nurses from two medical centers in Rafsanjan, Iran. Quota sampling was performed from August to November 2020. A demographic form, the Professional Quality of Life Scale (ProQOL), the Freiburg Mindfulness Questionnaire-Short Form (FMI-SF), and the Occupational Hardiness Questionnaire (OHQ) were used to collect data.

**Results:**

The mean age of the participants was 33.20 ± 6.85 years. The majority of the participants were female, married, and employed. Compassion Satisfaction (CS), Secondary Traumatic Stress (STS), and Burnout (BO) were all moderate among nurses. Hardiness was the best predictor of compassion satisfaction. Mindfulness was the best predictor of both secondary traumatic stress and burnout. Psychological hardiness and mindfulness had the greatest impact on nurses’ quality of professional life during the COVID-19 pandemic.

**Conclusion:**

Caring for COVID-19 patients may lead to BO, *CF*, and STS; identifying predictors of these can inform the development of interventions to mitigate or minimize BO, *CF*, and STS in nurses caring for these patients. Furthermore, in order to improve nurses’ quality of professional life, psychological hardiness, and mindfulness, necessary psychological programs and interventions should be designed and implemented.

## Introduction

COVID-19 became a major epidemic threat in China and around the world very quickly (Zakeri et al., 2021c). On January 30, 2020, the World Health Organization (WHO) declared COVID-19 a public health emergency of international concern (Zhu et al., 2020; Hossini Rafsanjanipoor et al., 2022). The COVID-19 pandemic has also put a lot of pressure on healthcare workers all over the world, causing anxiety and stress (Zakeri et al., 2021a). When compared to the general population, the prevalence of COVID-19 has caused many psychological and social problems in healthcare workers, particularly nurses (Zakeri et al., 2021d).

Nurses are on the front lines of preventing and treating COVID-19 and alleviating the suffering of COVID-19 patients. They are the most influential people in determining the capacity of healthcare facilities to handle COVID-19 cases (Buheji and Buhaid, 2020). Nurses, like other health-care workers, have been overwhelmed by rising demand, overwork, infection risk, the risk of disease transmission to their families, and voluntary isolation (Ortega-Galán et al., 2020). Nurses have experienced psychological symptoms such as fear, insecurity, and anxiety as a result of these difficult circumstances (Zakeri et al., 2021b).

Compassion fatigue (*CF*) is one of the psychological syndromes that the healthcare team faces when working in stressful and complex situations like the COVID-19 crisis. Compassion fatigue is a concept originally coined in the healthcare arena by Joinson (1992), when she noticed that emergency nurses had lost their “ability to nurture” (Roney and Acri, 2018). In fact, *CF* is exhaustion from dealing with other people’s suffering (Galiana et al., 2022) leading to a diminished ability to emphasize or feel compassion for others, often described as the negative cost of caring (Ruiz-Fernández et al., 2020).

Secondary traumatic stress (STS) has been described as a component of *CF* (Salimi et al., 2019). STS occurs as a reaction to secondary or indirect exposure to traumatic events experienced by another (Bride et al., 2007). People suffering from STS may experience symptoms such as irritability, poor concentration, anger, intrusive or recurring disturbing thoughts, and sleep disorders (Hinderer et al., 2014). Nurses are prone to STS because they are constantly exposed to others’ suffering, work in stressful environments, and spend their time and energy on others. These conditions reduce the empathetic capacity of nurses (Peters, 2018).

Burnout (BO) is closely related to STS (Ruiz-Fernández et al., 2020) and is regarded as a potential hazard in the workplace (Erkorkmaz et al., 2018). BO is a state of physical or emotional exhaustion that involves a sense of reduced accomplishment and loss of personal identity as a result of ongoing occupational difficulties (Ruiz-Fernández et al., 2020). Also, BO is a syndrome that can be experienced by healthcare providers in stressful situations. They are especially vulnerable because their work context is characterized by high-risk decisions, dealing with the public, and desires for compassion and sensitivity (Galiana et al., 2022). BO risk factors include environmental and work-related factors such as work overload and multiple employment, especially night shifts (Molina-Praena et al., 2018). BO and STS rates are reported to be more than 20% in nurses (Li et al., 2021).

Compassion satisfaction (Chung et al., 2009), which occurs when medical staff, including nurses, do their jobs properly, is a protective factor against STS. Furthermore, nurses’ satisfaction with their relationships with coworkers and a sense that what they are doing has social value (Roney and Acri, 2018). STS, CS, and BO are all parts of a larger concept known as “professional quality of life” (ProQol; Meyerson et al., 2020).

Existing literature reveals that nursing working environments differ from those of their counterparts in other countries, which may enhance their vulnerability to stress and mental distress. A study by Li and Xie (2020) has shown that ProQol transmitted the effect of personality to job satisfaction. Specifically, CS and BO mediated the positive effect of extraversion, agreeableness, conscientiousness, and openness on job satisfaction. STS has mediated the positive effect of extraversion on job satisfaction (Li and Xie, 2020). Also, BO and CS mediated the effect of anxiety upon depression, and STS mediated the effect of depression upon anxiety (Xie et al., 2021). Understanding these concepts is necessary to identify risk factors for mental health in nurses and to design interventions that promote nurses’ mental health (Li et al., 2021).

Mindfulness is one factor that has a positive impact on CS and BO. Mindfulness refers to awareness by paying attention to the moment without making judgments about what you are doing, which can vary from moment to moment and from person to person (Malakoutikhah et al., 2021a). The positive effects of mindfulness in reducing psychological problems of individuals have been reported in several studies (Dehghan et al., 2021; Malakoutikhah et al., 2021b). According to a study, a mindfulness-based stress reduction program significantly increased CS and decreased BO in emergency personnel (Ducar et al., 2020). Taylor et al. also demonstrated that mindfulness was a novel and distinct personal trait that could be applied to work patterns to reduce BO (Taylor and Millear, 2016). BO prevention has the potential to improve the quality and satisfaction of the services provided by healthcare providers (Habibi et al., 2014). It has been documented that mindfulness intervention in emergency medical service (EMS) providers has increased CS, attention to characteristics, and reduced BO (Ducar et al., 2020b). The study by Mou et al. showed that mindfulness can effectively predict the quality of nurses’ professional life (Mou et al., 2016).

In addition to mindfulness, another factor that affects quality of life and protects healthcare providers from job stress is psychological hardiness (Alipour Hamze Kandi and Zeinali, 2017). Psychological hardiness is defined as the ability to withstand stressful situations and focuses on human inner experience and mental perception (Moreno-Jiménez et al., 2014). Tenacious people have high control power and consider the challenges an opportunity for growth. Tenacious people know the importance, meaning, and values of who they are and what they do. Furthermore, they confirmed their role over that of others in solving problems and consider the change as a normal aspect of life (Maddi, 2006). One study found that nurses with higher levels of hardiness had a higher quality of life. Furthermore, psychological hardiness was one of the predictors of quality of life (Alipour Hamze Kandi and Zeinali, 2017). The results of Hatamipour et al.’s study showed that psychological hardiness had a positive and significant correlation with the quality of life among nurses (Hatamipour et al., 2017).

Factors related to the ProQol components in health care providers during the COVID-19 pandemic have been evaluated in a small number of studies, including three in Spain (Ortega-Galán et al., 2020; Ruiz-Fernández et al., 2020) and Italy (Buselli et al., 2020). Ortega-Galán et al. in Spain discovered that primary caregivers had slightly higher mean scores of STS and CS, while hospital caregivers had higher BO scores. There was a significant relationship between perceived stress and the three ProQol components (Ortega-Galán et al., 2020). In Spain, Ruiz-Fernández et al. found that physicians had higher STS and BO while nurses had higher CS. CS and perceived stress levels were equal regardless of the workplace (Ruiz-Fernández et al., 2020). Buselli et al. in Italy found that women had higher STS than men, while frontline workers and residents reported higher CSs than second-line staff and physicians (Buselli et al., 2020). These studies have been performed on various health professionals. Therefore, the present study only examines ProQol in nurses. In addition, the study of ProQol-related factors can be used in planning and interventions to promote it in nurses during the COVID-19 pandemic. Since no similar study has been performed on the ProQol of nurses in this pandemic in Iran, the current study aimed to investigate the relationships between ProQol, mindfulness, and psychological hardiness of nurses working in one hospital in Rafsanjan, Iran during the COVID-19 pandemic. Two research questions (RQs) are proposed: (1) Are ProQol, mindfulness and psychological hardiness correlated to one and other? (2) What are the predictors for ProQoL?”

## Materials and Methods

### Study Design, Setting, and Participants

The research participants included nurses working in one hospital in Rafsanjan, Iran, during the COVID-19 pandemic. This public hospital is the direct medical service center for COVID-19 patients in Rafsanjan city and has 350 active treatment beds. Data was collected from August to November 2020. Nurses with at least 1 year of work experience, working at least 1 month from their employment in the mentioned wards, nurses who provide care in the medical wards in contact with patients, and no managerial position (including head nurse, supervisor, or nurse manager) were all considered for inclusion. Nurses with a history of psychological disorders and chronic underlying diseases that lead to long-term drug use, as well as pregnant or lactating nurses, were excluded from the study.

### Sample Size and Sampling

Since the study population was limited, we used the census method for data collection. At the time of the study, 400 nurses were caring for COVID-19 patients in the mentioned hospital. Twenty nurses were not eligible to participate in the study. Of the rest, 141 nurses did not give consent to complete the surveys. Therefore, 239 nurses completed the surveys (the response rate = 62.89%). Calculations with G*Power software version 3.1.9.2. showed that 204 people would be needed to detect an effect size of 0.4 with a power of 80% and a value of *p* is 0.05 (for Pearson’s rho Correlation Coefficient test).

### Measures

#### Demographic and COVID-19 Information

Age, sex, level of education, marital status, number of children, work experience, type of employment, ward, type of shift, income, and monthly working hours were among the demographic information collected from the participants. Direct care of COVID-19 patients, infection with COVID-19, infection of relatives and friends, major concerns about the disease, and optimism about the disease outcome were all included in the COVID-19 information.

#### Professional Quality of Life Questionnaire

Stamm developed a 30-item self-reported measure called the Professional Quality of Life Questionnaire (Stamm, 2010). STS (10 items), CS (10 items), and BO (10 items) are three subscales of the ProQol. This questionnaire includes questions such as: “I am happy.,” “I get satisfaction from being able to [help] people.” and “I feel connected to others.” Each subscale contains 10 questions with a 5-point Likert scale (from never = 1 to always = 5). The questions (1, 4, 15, 17, and 29) are scored inversely. A score of 22 or less on all 3 subscales indicates a low level of STS, CS, and BO. A score of 23–41 indicates a moderate level of STS, CS, and BO, while a score of more than 42 indicates a higher level of STS, CS, and BO. An Iranian study confirmed the questionnaire’s validity and reliability, and Cronbach’s alpha was 0.80, 0.87, 0.76, and 0.57 for the whole questionnaire, CS, STS, and BO, respectively (Zakeri et al., 2020). In the present study, Cronbach’s alpha coefficient was 0.77 and that of CS, STS, and BO was 0.86, 0.82, and 0.72, respectively.

#### Freiburg Mindfulness Inventory-Short Form

The Freiburg Awareness Questionnaire was first developed by Walach et al. (2006) and consists of 30 questions. Later, they created a 14-item short form that is more appropriate for use in the general population. The shorter version is better for groups that are not familiar with Buddhism’s mindfulness practice, and it can be used in a variety of cultures. This questionnaire includes questions such as: “I am open to the experience of the present moment.” and “I sense my body‚ whether eating‚ cooking‚ cleaning or talking.” The questionnaire’s short form has two subscales: presence and acceptance. Questions are scored using a 4-point Likert scale (rarely = 1 to almost always = 4). Question 13 is scored inversely. The minimum score in this questionnaire is 14 and the maximum is 56. A higher score indicates higher mindfulness. In the study of GhasemiJobaneh et al. (2015), the total reliability coefficient of FMI-SF was 0.83 (GhasemiJobaneh et al., 2015). In the present study, the Cronbach’s alpha coefficient of the FMI-SF was 0.82.

#### Occupational Hardness Questionnaire

Moreno-Jimenez et al. developed the Job Hardiness Questionnaire. This questionnaire contains 3 subscales: control, challenge, and commitment in 17 questions for example “I involve myself seriously in what I do, because it is the best way to reach my own goals” and “Even when it supposes greater effort, I choose jobs that suppose a new experience for me.” Questions are graded on a 4-point Likert scale (disagree = 1 to agree = 4). People with a score of 45 or higher are hardy, while those with a lower score are not. Akbari Balotanbegan confirmed the OHQ in Iran using the internal consistency method and the Cronbach’s alpha coefficients for control, challenge, commitment, and the whole scale were 0.78, 0.81, 0.75, and 0.88, respectively (Akbari Balotanbegan et al., 2015). In the present study, Cronbach’s alpha coefficient was 0.76 for control, 0.71 for challenge, 0.70 for commitment, and 0.88 for the whole scale.

### Procedure

This study was conducted after acquiring the code of ethics from the ethics committee of Rafsanjan University of Medical Sciences. The reference number of the ethical approval is Ref. IR.RUMS.REC.1399.135. An informed consent form was attached to the questionnaire. Informed consent was obtained from nurses at the start of the study and before sampling, and they were informed about the study objectives, the confidentiality and anonymity of the information obtained, and the voluntary participation and withdrawal at any time. With the permission of the relevant authorities, the researcher went to the research settings and began sampling. Following access to the selected samples, the demographic and COVID-19 information questionnaire, the Professional Quality of Life Scale (ProQOL), the Freiburg Mindfulness Questionnaire (FMI-SF), and the Occupational Hardiness Questionnaire (OHQ) were distributed to the eligible samples.

### Data Analysis

SPSS-22 (IBM Corp., Armonk, NY, United States) was used to analyze the data. The basic demographic and clinical continuous data are shown as the mean and the standard deviation. These characteristics were compared using the Mann–Whitney, Kruskal–Wallis, and Chi-square tests. The Pearson correlation coefficient and multiple regression analysis were used to assess the relationship between ProQOL, mindfulness, and psychological hardiness. The values of *p* <0.05 were considered statistically significant.

## Results

### The Characteristics of the Participants

The mean age of the participants was 33.20 ± 6.85 (Min = 21 and Max = 54). The majority of the participants were female (172, 72.0%), married (182, 76.2%), employed (161, 67.4%) and had bachelor’s degrees (220, 92.1%; Table 1). The majority of the participants directly cared for the COVID-19 patients (150, 62.8%). Thirty-four participants were infected with COVID-19 (34, 14.2%). Sixty-nine participants had relatives/friends infected with the coronavirus (69, 28.9%). The most common concern about COVID-19 was family infection, which was expressed by 87.4 percent of the participants (Table 2).

**Table 1:** Demographic characteristics of the participants and their associations with Professional quality of life (*n* = 239).

Variables	Frequency (valid percent)	Professional quality of life					
		Compassion satisfaction		Secondary traumatic stress		Burnout	
		Mean ± SD	Statistical test (value of *p*)	Mean ± SD	Statistical test (value of *p*)	Mean ± SD	Statistical test (value of *p*)
**Gender**							
Male	67 (28.0)	37.38 ± 7.04	*t* = 2.08 (0.21)	27.71 ± 7.10	*t* = 1.62 (0.22)	23.55 ± 6.32	*t* = 1.15 (0.24)
Female	172 (72.0)	38.54 ± 6.31				26.58 ± 6.58			22.53 ± 5.57	
**Marital status**							
Unmarried/Widowed/Divorce	57 (23.8)	38.08 ± 6.94	*t* = 0.40 (0.86)	26.92 ± 6.16	*t* = 0.37 (0.47)	23.29 ± 6.08	*t* = 0.52 (0.96)
Married	182 (76.2)	38.26 ± 6.41		26.89 ± 6.92		22.67 ± 5.71	
**Number of children**							
0	76 (31.8)	38.23 ± 7.27	*F* = 0.49 (0.68)	26.53 ± 6.93	*F* = 0.31 (0.81)	23.06 ± 6.70	*F* = 0.17 (0.91)
1	55 (23.0)	37.78 ± 7.34		27.34 ± 6.65		23.01 ± 5.64	
2	81 (33.9)	38.04 ± 5.55		27.02 ± 6.78		22.77 ± 5.29	
3≤	27 (11.3)	39.59 ± 5.33		26.62 ± 6.52		21.85 ± 4.91	
**Educational level**							
Bachelor	220 (92.1)	38.30 ± 6.63	*Z* = −0.83 (0.40)	26.74 ± 6.75	*Z* = −0.43 (0.66)	22.75 ± 5.82	*Z* = −1.25 (0.20)
Masters	19 (7.9)	37.31 ± 5.19		28.73 ± 6.40		23.52 ± 5.61	
**Income (Million Riyal)**							
<3	27 (11.3)	39.40 ± 7.63	*F* = 0.51 (0.60)	23.70 ± 6.83	*F* = 2.01 (0.13)	20.74 ± 5.52	*F* = 3.58 (0.03)
3–5	176 (73.6)	38.03 ± 6.43		27.22 ± 6.77		23.13 ± 5.84	
>5	36 (15.1)	38.22 ± 6.20		27.72 ± 5.95		22.83 ± 5.58	
**Type of employment**							
Hired	161 (67.4)	38.09 ± 6.30	*t* = 0.63 (0.66)	27.62 ± 6.59	*t* = 2.01 (0.04)	23.34 ± 5.67	*t* = 2.42 (0.02)
Contract recruiters^a^/Committed^b^	78 (32.6)	38.48 ± 7.00		25.39 ± 6.81		21.74 ± 5.94	
**Work experience (yr.)**							
>5	84 (35.1)	38.34 ± 7.49	*H* = 2.80 (0.42)	26.14 ± 6.70	*H* = 2.65 (0.44)	22.25 ± 6.36	*H* = 2.56 (0.46)
5–10	67 (28.0)	37.46 ± 6.03		27.26 ± 6.77		23.68 ± 5.53	
11–15	38 (15.9)	37.76 ± 5.98		26.57 ± 6.76		23.00 ± 5.26	
>15	50 (21.0)	39.38 ± 5.80		27.92 ± 6.76		22.48 ± 5.53	
**Ward**							
Critical/intensive	86 (36.0)	38.38 ± 6.46	*F* = 2.11 (0.10)	27.08 ± 6.55	*F* = 2.75 (0.04)	22.68 ± 5.51	*F* = 0.93 (0.42)
Emergency	65 (27.2)	38.47 ± 7.16		27.38 ± 6.97		23.24 ± 6.46	
Medical	59 (24.7)	36.69 ± 5.96		27.05 ± 7.19		23.83 ± 5.73	
Others	29 (12.1)	40.27 ± 5.95		24.96 ± 5.71		20.20 ± 4.47	
**Shift**							
Fixed	23 (9.6)	38.21 ± 8.14	*t* = 1.20 (0.99)	27.26 ± 7.49	*t* = 1.48 (0.90)	22.95 ± 5.36	*t* = 1.34 (0.78)
Not fixed	216 (90.4)	38.22 ± 6.36		26.86 ± 6.67		22.80 ± 5.85	
**Mandatory working hours per month**							
<150	44 (18.4)	39.72 ± 7.29	*F* = 1.20 (0.30)	28.18 ± 7.44	*F* = 0.06 (0.97)	22.77 ± 5.81	*F* = 1.08 (0.35)
150–160	107 (44.8)	37.53 ± 5.91		26.66 ± 6.34		22.75 ± 5.48	
161–170	49 (20.5)	38.14 ± 4.91		25.81 ± 6.37		23.14 ± 5.57	
>170	39 (16.3)	38.51 ± 8.64		27.46 ± 7.35		22.64 ± 6.99	

*Data were presented as number (%). t, Independent t test; F, Analysis of variance test; Z, Mann Whitney U test; H, Kruskal Wallis test.*

a Annually contracted with payment similar to hired nurses. ^b^It is obligatory to work for government for 2 years at a lower rate of pay.

**Table 2:** Information related to COVID-19 disease of the participants and their associations with professional quality of life (*n* = 239).

Variables	Frequency (valid percent)	Professional quality of life					
		Compassion satisfaction		Secondary traumatic stress		Burnout	
		Mean ± SD	Statistical test (value of *p*)	Mean ± SD	Statistical test (value of *p*)	Mean ± SD	Statistical test (value of *p*)
**Caring directly of positive COVID-19 patients**							
Yes	150 (62.8)	38.42 ± 6.61	*t* = 0.44 (0.54)	26.82 ± 7.12	*t* = −0.13 (0.89)	22.78 ± 6.25	*t* = −0.23 (0.81)
No	89 (37.2)	37.88 ± 6.41				27.03 ± 6.06			22.88 ± 4.96	
**Infected with COVID-19**							
Yes	34 (14. 2)	38.08 ± 5.94	*Z* = −0.76 (0.44)	27.76 ± 6.00	*Z* = −0.10 (0.92)	22.97 ± 6.85	*Z* = −0.48 (0.63)
No	205 (85.8)	38.24 ± 6.63		26.75 ± 6.85		22.79 ± 5.62	
**Relatives/ friends infected with COVID-19**							
Yes	69 (28.9)	39.34 ± 6.06	*t* = 0.66 (0.09)	27.42 ± 6.74	*t* = 0.16 (0.47)	22.39 ± 5.77	*t* = 0.05 (0.44)
No	170 (71.1)	37.76 ± 6.67		26.68 ± 6.74		22.99 ± 5.81	
**The most common concern about COVID-19**							
My family getting sick	209 (87.4)	36.43 ± 7.10	*t* = 0.24 (0.10)	27.70 ± 7.83	*t* = 1.51 (0.83)	23.03 ± 5.23	*t* = 0.07 (0.48)
Others^‡^	30 (12.6)	38.47 ± 6.42		26.78 ± 6.57		22.78 ± 5.88	
**Being optimistic about the outcome of COVID-19**							
Yes	132 (55.2)	38.91 ± 6.66	*t* = 1.50 (0.06)	25.78 ± 6.13	*t* = −2.73 (0.007)	21.90 ± 5.83	*t* = −2.89 (0.004)
No	107 (44.8)	37.36 ± 6.29		28.28 ± 7.21		23.94 ± 5.58	

*Data were presented as number (%). t, Independent t test; Z, Mann Whitney U test.*

‡ Other reasons (death, hospitalization, my getting sick, my friends getting sick and no concern).

**Figure 1:**
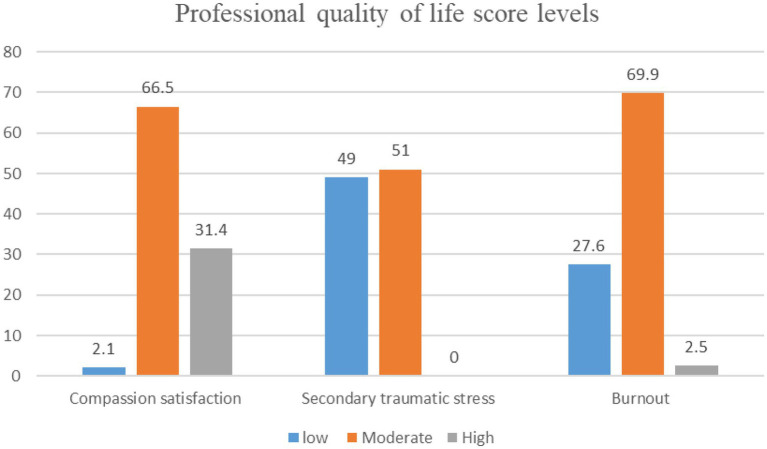
The level of professional quality of life during COVID-19.

The mean score of CS was 38.22 ± 6.53. The mean scores of STS and BO were 22.82 ± 5.73 and 26.89 ± 6.73, respectively. Among the ProQol subscales, the CS had the highest score, and the STS had the lowest score. The majority of nurses had moderate levels of CS (159, 66.5%), STS (122, 51.0%), and BO (167, 69.9%; Figure 1).

The mean score of hardiness was 50.09 ± 7.24. Of 239 participants, 197 (82.4%) had hardiness. The mean score of mindfulness was 32.29 ± 6.32 (Min = 18, Max = 53; Table 3).

**Table 3:** The correlation between the underlying factors of ProQOL, mindfulness, and hardiness among nurse (*n* = 239).

Variable	Mean	SD	Pearson’s rho Correlation Coefficient				
			CS	STS	BO	Hardiness	Mindfulness
1. CS (ProQOL)	38.22	6.53					
2. STS (ProQOL)	22.82	5.73	−0.63^***^				
3. BO (ProQOL)	26.89	6.73	−0.15	0.64^***^			
4. Hardiness	50.09	7.24	0.52^***^	−0.34^***^	−0.09		
5. Mindfulness	35.29	6.32	0.41^***^	−0.46^***^	−0.30^***^	0.46^***^	

*SD, Standard deviation; ProQOL, Professional Quality of Life; CS, compassion satisfaction; STS, secondary traumatic stress; BO, burnout*.

*** *p < 0.001*.

A positive significant correlation was observed between CS, hardiness (*r* = 0.52), and mindfulness (*r* = 0.41). STS had a negative significant correlation with hardiness (*r* = −0.34) and mindfulness (*r* = −0.46). Similarly, a significant correlation was found between BO and mindfulness (*r* = −0.30). In addition, there was a positive, significant correlation between hardiness and mindfulness (*r* = 0.46; Table 3).

### The Correlation Between ProQol, Mindfulness, and Psychological Hardiness

Bivariate analysis showed no association between CS and background characteristics. The mean score of STS had a significant association with ward and being optimistic about the outcome of COVID-19 (*p* < 0.05). In addition, variables of income (*p* = 0.03) and type of employment (*p* = 0.02) were significantly associated with the mean score of BO. In addition, BO had a significant association with income, type of employment, and being optimistic about the outcome of COVID-19 (*p* < 0.05; Tables 1, 2).

### The Predictors for ProQol

Multiple regression models were tested to explore how demographic variables, mindfulness, and hardiness predicted CS, STS, and BO. As shown in Table 4, 35% of the variance of CS is predicted by hardiness, mindfulness, concern about family and others getting sick with COVID-19, type of employment, gender, ward, and job history (Adjusted *R*^2^ = 35%), and the best predictor was hardiness (*p* < 0.001). Mindfulness, type of employment, hardiness, gender, and number of children predicted 29% of the variance of STS (Adjusted *R*^2^ = 29%) and the best predictor was mindfulness (*p* < 0.001). Finally, mindfulness, type of employment, and gender predicted 13% of the variance of BO (Adjusted *R*^2^ = 13%) and, based on β, the best predictor was mindfulness (*p* < 0.001).

**Table 4:** Multiple regression analysis summary for predictors of ProQol among nurse (*n* = 239).

Variable		B	SE^‡^	Beta	*t*	*p*	95% Confidence interval for B	Adjusted *R*^2^
Compassion satisfaction	Constant	−3.68	4.17		−0.88	0.38	−11.90–4.53	35%
	Hardiness	0.39	0.05	0.44	7.40	<0.001	0.29–0.50	
	Mindfulness	0.24	0.06	0.24	4.02	<0.001	0.12–0.37	
	Gender	2.31	0.77	0.16	2.98	0.003	0.78–3.84	
	Type of employment	1.78	0.82	0.12	2.14	0.03	0.14–3.41	
	Concern about getting sick with COVID-19	2.19	1.02	0.11	2.13	0.03	0.16–4.22	
	Ward	0.67	0.32	0.10	2.06	0.04	0.03–1.32	
	Job history	0.58	0.34	0.10	1.71	0.09	−0.08–1.25	
Secondary traumatic stress	Constant	51.74	3.15		16.38	<0.001	45.52–57.96	29%
	Mindfulness	−0.38	0.05	−0.42	−6.83	<0.001	−0.50–−0.27	
	Type of employment	−2.91	0.74	−0.23	−3.91	<0.001	−4.38–−1.44	
	Hardiness	−0.13	0.04	−0.17	−2.80	0.005	−0.23–−0.41	
	Gender	−1.98	0.72	−0.15	−2.74	0.007	−3.40–−0.55	
	Number of children	−0.80	0.34	−0.14	−2.33	0.02	−1.48–−0.12	
Burnout	Constant	46.54	3.31		14.03	<0.001	40.01–53.08	13%
	Mindfulness	−0.35	0.06	−0.33	−5.43	<0.001	−0.48–−0.22	
	Type of employment	−2.67	0.87	−0.18	−3.05	0.003	−4.40–−0.95	
	Gender	−2.11	0.92	−0.14	−2.30	0.02	−3.93–−0.30	

*Data were presented as multiple regression analysis. Only significant results were shown; CI, Confidence intervals for B; ProQOL, Professional quality of life; Gender (male = 1 and female = 2); Job history (> 5 = 1, 5–10 = 2, 11–15 = 3 and > 15 = 4); Type of employment (Hired = 1 and others contract recruiters/Committed = 2); Ward (Critical/intensive = 1, Emergency = 2, Medical = 3, and Others = 4)*.

‡ *Standard error*.

## Discussion

### The Correlation Between ProQol, Mindfulness, and Psychological Hardiness

According to the study results, the variables that had a significant effect on CS and STS were hardiness, mindfulness, gender, and type of employment. Nurses with higher CS and lower STS were hardier and more mindful. According to the study results, increasing mindfulness was also associated with a decrease in BO. Phillips (2011) investigated hardiness as a possible defense against STS (Phillips, 2011). Another study found hardiness to be one of the predictors of nurses’ quality of life (Alipour Hamze Kandi and Zeinali, 2017). Hotchkiss (2018) found that mindful self-care was positively correlated with CS and negatively correlated with STS and BO in nurses providing hospice care, which is consistent with these results (Hotchkiss, 2018).

The results of this study support the expected relationships between the three ProQol subscales: STS has a negative relationship with CS and a positive relationship with BO. Theoretically, it is possible to deduce that methods, which increase CS, can reduce STS.

Results of Cuartero-Castañer et al. on the prevalence of COVID-19 showed healthcare workers have an average quality of life with high levels of compassion satisfaction and average levels of compassion fatigue and burnout. Data also indicated that the sample frequently engaged in self-care practices and had high levels of work engagement (Cuartero-Castañer et al., 2021). Some studies suggest that having experienced adversity in the past may predispose individuals toward compassion (Lim and DeSteno, 2016). Nurses’ BO and STS risk factors should be assessed and specialty-specific interventions may be considered to improve CS while reducing BO and STS among nurses (Lykins et al., 2021). Nursing managers should implement practical and comprehensive plans to increase nurses’ CS and reduce BO and STS (Zakeri et al., 2020).

Female nurses had higher CS and lower STS and BO in this study. Gonzalez et al. (2019) found that among trauma responders in Michigan, gender had no significant effect on any of the ProQol subscales (Gonzalez et al., 2019). Another study found only a significant relationship between gender and CS, but no relationship between gender and STS or BO (Roney and Acri, 2018). This difference can be due to differences in the research population, organizational and managerial climates, professional workload, and differences in nurses’ incomes in various communities. Furthermore, given the contradictory findings regarding the relationship between gender and the ProQol subscales, future research should focus on any potential relationship between these subscales and gender.

### The Predictors for ProQol

The study results revealed that the type of employment had a positive relationship with CS but a negative relationship with STS and BO. In Iran, the majority of nurses in the present study were hired due to their higher work experience and, consequently, older age. Results of Sacco et al. (2015) study also showed that CS increased with age (Sacco et al., 2015). In contrast, as people get older, their ability to cope with and adapt to stressful events related to patients decreases due to individual and personality changes. Therefore, it is possible to justify the inverse relationship between the types of employment, STS, and BO. One study also confirmed a positive relationship between age and STS (Wentzel and Brysiewicz, 2018).

Furthermore, the study results revealed a relationship between the type of ward and CS. In contrast to this finding, a study found no relationship between the ward and ProQol (Hooper et al., 2010). This discrepancy could be due to the questionnaire’s self-reporting nature and cultural differences. These contradictory results also point to the need for more research.

According to the results of the current study, nurses with a higher CS are more concerned about the illness of their family and relatives. The concern was not identified as a factor influencing CS in the studies. However, the authors hypothesized that CS contains the concept of concern about others that can at least partially justify the existence of this relationship in the context of COVID-19. Humanistic nursing practice constitutes the cornerstone of the nursing profession and strengthens the value of altruism among clinical nurses for the greater good of patients (Alavi et al., 2017). Altruistic nurses support the well-being of patients within their professional capacity and engage in caring acts motivated by concerns for others (Swank et al., 2013). However, some studies on the prevalence of coronavirus have shown that medical staff are concerned about family and acquaintances with coronavirus, and this is mostly due to nursing care and nurses’ close relationships with coronavirus patients (Zakeri et al., 2021a). However, understanding why nurses are concerned and what causes them to be concerned, especially during crises, needs to be looked into in future studies.

Another factor associated with STS, according to the results, is the number of children. The greater the number of children, the lower the STS. According to one study, having a child lowers the risk of STS because children provide emotional support and prevent people from concentrating on workplace stressors (Haik et al., 2017).

Another highlight of the study was the significant association between STS and BO with being optimistic about the outcome of COVID-19. However, the effect of optimism level on the STS and BO of nurses was not measured in this study. These results could be consistent with the study by Cruz et al. (2018). Their study showed that optimism among nurses is significantly associated with a better quality of life (Cruz et al., 2018). These findings are important for the nursing profession in the COVID-19 pandemic because nurses often experience increased job stressors and heavy workloads in the pandemic; a positive thinking approach will enable them to cope with the stressful experiences (Motamed-Jahromi et al., 2017). Therefore, when COVID-19 disease starts spreading, it is important to think about the best ways to help nurses deal with their anxiety and stress, like training programs and counseling (Zakeri and Dehghan, 2021).

There are limitations to this study that should be considered when using the results. This study only included nurses working in one hospital affiliated with Rafsanjan University of Medical Sciences and did not address other healthcare workers. As a result, it is suggested that additional research be conducted, taking into account all healthcare workers and comparing the results with the quality of professional life among them. Furthermore, the study design was cross-sectional, and the study data were collected using self-reported questionnaires, which may have had an impact on the study results. Finally, as our study population was limited, we selected a large effect size of 0.4 for the sample size. It is recommended that future studies use a smaller effect size and, therefore, a larger sample. Calculations with G*Power software version 3.1.9.2. show that 510 people would be needed to detect an effect size of 0.25 with a power of 80% and a *p* value of 0.05 (for Pearson’s rho Correlation Coefficient test).

## Conclusion

According to the results of this study, all subscales of ProQol, including CS, STS, and BO, were moderate in nurses. Some sociodemographic variables, such as gender, number of children, type of employment, ward, and work experience, correlated with the subscales. Mindfulness and psychological hardiness were associated with almost all subscales of ProQol. Therefore, it is suggested that researchers, managers, and nursing policy makers pay more attention to the factors affecting ProQol and take an effective step in promoting the ProQol of nurses by providing and implementing appropriate solutions such as training and counseling programs to increase psychological hardiness and mindfulness. In addition, according to the prevalence of STS and BO, nurses’ conditions and the pattern of the COVID-19 epidemic in each hospital must be concisely considered to allocate proper medical treatments and management of the patients, as well as proper allocation of nurses to the COVID centers.

## Data Availability Statement

The raw data supporting the conclusions of this article will be made available by the authors, without undue reservation.

## Ethics Statement

The studies involving human participants were reviewed and approved by Rafsanjan University of Medical Sciences. The patients/participants provided their written informed consent to participate in this study.

## Author Contributions

MZ, FG-H, EK, SH, and MD developed the study idea and protocol. MZ and MA supervised the study sampling. EK, HG, HP, MM, and AZ did the sampling. MD analyzed the data. MZ, FG-H, SH, and MA wrote the first draft of the manuscript. All authors contributed to the article and approved the submitted version.

## Funding

This study is part of the research project no. IR.RUMS.99140.

## Conflict of Interest

The authors declare that the research was conducted in the absence of any commercial or financial relationships that could be construed as a potential conflict of interest.

## Publisher’s Note

All claims expressed in this article are solely those of the authors and do not necessarily represent those of their affiliated organizations, or those of the publisher, the editors and the reviewers. Any product that may be evaluated in this article, or claim that may be made by its manufacturer, is not guaranteed or endorsed by the publisher.

## References

[ref1] Akbari Balotanbegan A. Rezaei A. Far M. Najafi M. Akbari Balootbangan I. (2015). Psychometric properties of occupational hardiness questionnaire short form among nurses. Iran J. Nurs. 28, 55–65. doi: 10.29252/ijn.28.93.94.55

[ref2] Alavi A. Zargham-Boroujeni A. Yousefy A. Bahrami M. (2017). Altruism, the values dimension of caring self-efficacy concept in Iranian pediatric nurses. J. Educ. Health Promot. 6:8. doi: 10.4103/jehp.jehp_4142_4114 28546973 PMC5433645

[ref3] Alipour Hamze Kandi N. Zeinali A. (2017). Relationship between personality characteristics, internal locus of control, psychological hardiness and nurses’ quality of life [original article]. J. Res. Develop. Nursing and Midwifery 14, 8–15. doi: 10.29252/jgbfnm.14.1.8

[ref4] Bride B. E. Radey M. Figley C. R. (2007). Measuring compassion fatigue. Clin. Soc. Work. J. 35, 155–163. doi: 10.1007/s10615-10007-10091-10617

[ref5] Buheji M. Buhaid N. (2020). Nursing human factor during COVID-19 pandemic. Intern. J. Nursing 10, 12–24. doi: 10.5923/j.nursing.20201001.02

[ref6] Buselli R. Corsi M. Baldanzi S. Chiumiento M. Del Lupo E. Dell Oste V. Bertelloni C. A. Massimetti G., . (2020). Professional quality of life and mental health outcomes among health care workers exposed to Sars-Cov-2 (Covid-19). Int. J. Environ. Res. Public Health, 17, 6180. doi: 10.3390/ijerph17176180, PMID: 32858810 PMC7504107

[ref7] Chung C. Y. Koprich J. B. Siddiqi H. Isacson O. (2009). Dynamic changes in presynaptic and axonal transport proteins combined with striatal neuroinflammation precede dopaminergic neuronal loss in a rat model of AAV alpha-synucleinopathy. J. Neurosci. 29, 3365–3373. doi: 10.1523/jneurosci.5427-08.2009, PMID: 19295143 PMC2693917

[ref8] Cruz J. Cabrera D. Hufana O. Alquwez N. Almazan J. (2018). Optimism, proactive coping and quality of life among nurses: A cross-sectional study. J. Clin. Nurs. 27, 2098–2108. doi: 10.1111/jocn.14363, PMID: 29603804

[ref9] Cuartero-Castañer M. E. Hidalgo-Andrade P. Cañas-Lerma A. J. (2021). Professional quality of life, engagement, and self-care in healthcare professionals in Ecuador during the COVID-19 pandemic. Health 9:515. doi: 10.3390/healthcare9050515, PMID: 33946629 PMC8146458

[ref10] Dehghan M. Namjoo Z. Mohammadi Akbarabadi F. Fooladi Z. Zakeri M. A. (2021). The relationship between anxiety, stress, spiritual health, and mindfulness among patients undergoing hemodialysis: A survey during the COVID-19 outbreak in Southeast Iran. Health Sci. Repor. 4:e461. doi: 10.1002/hsr1002.1461, PMID: 34938901 PMC8670730

[ref11] Ducar D. M. Penberthy J. K. Schorling J. B. Leavell V. A. Calland J. F. (2020). Mindfulness for healthcare providers fosters professional quality of life and mindful attention among emergency medical technicians. Exp. Dermatol. 16, 61–68. doi: 10.1016/j.explore.2019.07.015, PMID: 31471216

[ref12] Erkorkmaz U. Dogu O. Cinar N. (2018). The relationship between burnout, self-esteem and professional life quality of nurses. J. College of Physicians and Surgeons Pakistan 28, 549–553. doi: 10.29271/jcpsp.2018.07.549, PMID: 29950262

[ref13] Galiana L. Sansó N. Muñoz-Martínez I. Vidal-Blanco G. Oliver A. Larkin P. J. (2022). Palliative care professionals’ inner life: exploring the mediating role of self-compassion in the prediction of compassion satisfaction, compassion fatigue, burnout and wellbeing. J. Pain Symptom Manag. 63, 112–123. doi: 10.1016/j.jpainsymman.2021.07.004, PMID: 34271144

[ref14] GhasemiJobaneh R. Arabzadeh M. JaliliNikoo S. MohammadAlipoor Z. Mohsenzadeh F. (2015). Survey the validity and reliability of the persian version of short form of freiburg mindfulness inventory. J. Rafsanjan Univer. Medical Sci. 14, 137–150.

[ref15] Gonzalez T. C. Burnett H. Helm H. Edwards L. (2019). An examination of resilience, compassion fatigue, burnout, and compassion satisfaction between men and women among trauma responders. N. Am. J. Psychol. 21, 1–20.

[ref16] Habibi H. Mooghali A. Bagheri Lankarani K. Habibi F. (2014). Relationship between nurses' job satisfaction and burnout with patients satisfaction in shiraz, 2012. Journal of hayat 20, 30–42.

[ref17] Haik J. Brown S. Liran A. Visentin D. Sokolov A. Zilinsky I.. (2017). Burnout and compassion fatigue: prevalence and associations among Israeli burn clinicians. Neuropsychiatr. Dis. Treat. 13, 1533–1540. doi: 10.2147/NDT.S133181, PMID: 28670122 PMC5478274

[ref18] Hatamipour K. Hoveida F. Rahimaghaee F. Babaeiamiri N. Ashoori J. (2017). The nurses' quality of life based on burnout, perceived social support and psychological hardiness. J. Res. Develop. Nursing and Midwifery 14, 22–28. doi: 10.29252/jgbfnm.29214.29251.29222

[ref19] Hinderer K. A. VonRueden K. T. Friedmann E. McQuillan K. A. Gilmore R. Kramer B.. (2014). Burnout, compassion fatigue, compassion satisfaction, and secondary traumatic stress in trauma nurses. J. trauma nursing | JTN 21, 160–169. doi: 10.1097/jtn.0000000000000055, PMID: 25023839

[ref20] Hooper C. Craig J. Janvrin D. R. Wetsel M. A. Reimels E. (2010). Compassion satisfaction, burnout, and compassion fatigue among emergency nurses compared with nurses in other selected inpatient specialties. J. Emerg. Nurs. 36, 420–427. doi: 10.1016/j.jen.2009.11.027, PMID: 20837210

[ref21] Hossini Rafsanjanipoor S. M. Zakeri M. A. Dehghan M. Kahnooji M. Sanji Rafsanjani M. Ahmadinia H.. (2022). Iranian psychosocial status and its determinant factors during the prevalence of COVID-19 disease. Psychol. Health Med. 27, 30–41. doi: 10.1080/13548506.13542021.11874438 33486996

[ref22] Hotchkiss J. T. (2018). Mindful self-care and secondary traumatic stress mediate a relationship Between compassion satisfaction and burnout risk Among hospice care professionals. Am. J. Hosp. Palliat. Med. 35, 1099–1108. doi: 10.1177/1049909118756657, PMID: 29482332

[ref400] Joinson C. (1992). Coping with compassion fatigue. Nursing 22, 116–118., PMID: 1570090

[ref23] Li W. W. West C. Xie G. (2021). The reflective risk assessment model of professional quality of life in Chinese nurses. J. Nurs. Manag. 29, 767–775. doi: 10.1111/jonm.13217, PMID: 33249646 PMC7753301

[ref24] Li W. W. Xie G. (2020). Personality and job satisfaction among Chinese health practitioners: The mediating role of professional quality of life. Health Psychol. Open 7:2055102920965053. doi: 10.1177/2055102920965053, PMID: 33178439 PMC7592332

[ref25] Lim D. DeSteno D. (2016). Suffering and compassion: The links among adverse life experiences, empathy, compassion, and prosocial behavior. Emotion 16, 175–182. doi: 10.1037/emo0000144, PMID: 26751630

[ref26] Lykins A. B. Seroka N. W. Mayor M. Seng S. Higgins J. T. Okoli C. T. (2021). Compassion satisfaction, burnout, and secondary traumatic stress Among nursing staff at an Academic Medical Center: A cross-sectional analysis. J. Am. Psychiatr. Nurses Assoc. 10783903211066125. doi: 10.1177/10783903211066125 34931579

[ref27] Maddi S. R. (2006). Hardiness: The courage to grow from stresses. J. Posit. Psychol. 1, 160–168. doi: 10.1080/17439760600619609

[ref28] Malakoutikhah A. Zakeri M. A. Derakhtanjani A. S. Dehghan M. (2021a). Anxiety, anger, and mindfulness as predictors of general health in the general population during COVID-19 outbreak: A survey in Southeast Iran. J. Community Psychol. 50, 916–927. doi: 10.1002/jcop.22690, PMID: 34409604 PMC8426806

[ref29] Malakoutikhah A. Zakeri M. A. Salehi Derakhtanjani A. Dehghan M. (2021b). The psychometric properties of the relaxation/meditation/mindfulness (RMM) tracker t inventory in an Iranian population. Biomed. Res. Int. 2021, 1–10. doi: 10.1155/2021/2998916, PMID: 35005015 PMC8741359

[ref30] Meyerson J. Gelkopf M. Eli I. Uziel N. (2020). Burnout and professional quality of life among Israeli dentists: the role of sensory processing sensitivity. Int. Dent. J. 70, 29–37. doi: 10.1111/idj.12523, PMID: 31560417 PMC9379207

[ref31] Molina-Praena J. Ramirez-Baena L. Gómez-Urquiza J. L. Cañadas G. R. De la Fuente E. I. Cañadas-De la Fuente G. A. (2018). Levels of burnout and risk factors in medical area nurses: A Meta-analytic study. Int. J. Environ. Res. Public Health 15:2800. doi: 10.3390/ijerph15122800, PMID: 30544672 PMC6313576

[ref32] Moreno-Jiménez B. Rodríguez-Muñoz A. Hernández E. G. Blanco L. M. (2014). Development and validation of the occupational hardiness questionnaire. Psicothema 26, 207–214. 24755022 10.7334/psicothema2013.49

[ref33] Motamed-Jahromi M. Fereidouni Z. Dehghan A. (2017). Effectiveness of positive thinking training program on Nurses' quality of work life through smartphone applications. Intern. scholarly res. notices 2017:4965816. doi: 10.1155/2017/4965816, PMID: 28589174 PMC5446857

[ref34] Mou H. Chen X. Sun Y. Yan T. Li J. (2016). The impact of mindfulness on nurses’ professional quality of life. Chinese J. Practical Nursing 36, 206–210. doi: 10.3760/cma.j.issn.1672-7088.2016.03.016

[ref35] Ortega-Galán Á. M. Ruiz-Fernández M. D. Lirola M.-J. Ramos-Pichardo J. D. Ibáñez-Masero O. Cabrera-Troya J., (.) (2020). Professional Quality of Life and Perceived Stress in Health Professionals Before COVID-19. Spain: Primary and Hospital Care. Healthcare. 10.3390/healthcare8040484 PMC7711881 33202750

[ref36] Peters E. (2018). Compassion fatigue in nursing: A concept analysis. Nurs. Forum 53, 466–480. doi: 10.1111/nuf.12274, PMID: 29962010

[ref37] Phillips J. (2011). Hardiness as a defense against compassion fatigue and burnout. J. Emerg. Nurs. 37:125. doi: 10.1016/j.jen.2010.10.012, PMID: 21397123

[ref38] Roney L. N. Acri M. C. (2018). The cost of caring: An exploration of compassion fatigue, compassion satisfaction, and job satisfaction in pediatric nurses. J. Pediatr. Nurs. 40, 74–80. doi: 10.1016/j.pedn.2018.01.016, PMID: 29402658

[ref39] Ruiz-Fernández M. D. Pérez-García E. Ortega-Galán Á. M. (2020). Quality of life in nursing professionals: burnout, fatigue, and compassion satisfaction. Int. J. Environ. Res. Public Health 17:1253. doi: 10.3390/ijerph17041253, PMID: 32075252 PMC7068555

[ref40] Sacco T. L. Ciurzynski S. M. Harvey M. E. Ingersoll G. L. (2015). Compassion satisfaction and compassion fatigue Among critical care nurses. Crit. Care Nurse 35, 32–42. doi: 10.4037/ccn2015392 26232800

[ref41] Salimi S. Pakpour V. Rahmani A. Wilson M. Feizollahzadeh H. (2019). Compassion satisfaction, burnout, and secondary traumatic stress Among critical care nurses in Iran. J. Transcult. Nurs. 31, 59–66. doi: 10.1177/1043659619838876, PMID: 30957715

[ref500] Stamm B. (2010). The concise manual for the professional quality of life scale. ID: ProQOL.org., PMID: 29962010

[ref42] Swank J. M. Ohrt J. H. Robinson E. M. (2013). A qualitative exploration of counseling students' perception of altruism. The J. Humanistic Counseling 52, 23–38. doi: 10.1002/j.2161-1939.2013.00030.x

[ref43] Taylor N. Z. Millear P. M. R. (2016). The contribution of mindfulness to predicting burnout in the workplace. Personal. Individ. Differ. 89, 123–128. doi: 10.1016/j.paid.2015.10.005

[ref501] Walach H. Buchheld N. Buttenmüller V. Kleinknecht N. Schmidt S. (2006). Measuring mindfulness—the Freiburg mindfulness inventory (FMI). Pers. Individ. Differ. 40, 1543–1555., PMID: 34237163

[ref44] Wentzel D. Brysiewicz P. (2018). A survey of compassion satisfaction, burnout and compassion fatigue in nurses practicing in three oncology departments in Durban, South Africa. Intern. j. Africa nursing sci. 8, 82–86. doi: 10.1016/j.ijans.2018.03.004

[ref45] Xie G. Li W. McDermott B. (2021). Professional quality of life as potential mediators of the association between anxiety and depression among Chinese health-care clinicians. The Intern. J. Psychiatry in Med. 56, 83–96. doi: 10.1177/0091217420913395, PMID: 32220213

[ref46] Zakeri M. A. Bazmandegan G. Ganjeh H. Zakeri M. Mollaahmadi S. Anbariyan A.. (2020). Is nurses’ clinical competence associated with their compassion satisfaction, burnout and secondary traumatic stress? A cross-sectional study. Nursing Open 8, 354–363. doi: 10.1002/nop1002.1636, PMID: 33318843 PMC7729795

[ref47] Zakeri M. A. Dehghan M. (2021). The role of continuing education in protecting nurses against COVID-19 infection. J. Occup.l Health and Epidemiol. 10, 64–66. doi: 10.52547/johe.52510.52542.52564

[ref48] Zakeri M. A. Dehghan M. Heidari F. G. Pakdaman H. Mehdizadeh M. Ganjeh H.. (2021a). Mental health outcomes among health-care workers during the COVID-19 outbreak in Iran. Ment. Health Rev. J. 26, 152–160. doi: 10.1108/MHRJ-1110-2020-0075

[ref49] Zakeri M. A. Hossini Rafsanjanipoor S. M. Zakeri M. Dehghan M. (2021b). The relationship between frontline nurses' psychosocial status, satisfaction with life and resilience during the prevalence of COVID-19 disease. Nurs. Open 8, 1829–1839. doi: 10.1002/nop1002.1832 33675182 PMC8186693

[ref50] Zakeri M. A. Maazallahi M. Ehsani V. Dehghan M. (2021c). Iranian psychosocial status during and after COVID-19 outbreak mandatory quarantine: A cross-sectional study. J. Community Psychol. 49, 2506–2516. doi: 10.1002/jcop.22647, PMID: 34237163

[ref51] Zakeri M. A. Rafsanjanipoor S. M. H. Sedri N. Kahnooji M. Rafsanjani M. S. Zakeri M.. (2021d). Psychosocial status during the prevalence of COVID-19 disease: the comparison between healthcare workers and general population. Curr. Psychol. 40, 6324–6332. doi: 10.1007/s12144-12021-01582-12141 33746463 PMC7963465

[ref52] Zhu Z. Xu S. Wang H. Liu Z. Wu J. Li G.. (2020). COVID-19 in Wuhan: immediate psychological impact on 5062 health workers. MedRxiv:5338. doi: 10.1101/2020.1102.1120.20025338

